# Circulating Mucosal-Associated Invariant T Cells in a Large Cohort of Healthy Chinese Individuals From Newborn to Elderly

**DOI:** 10.3389/fimmu.2019.00260

**Published:** 2019-02-19

**Authors:** Pengcheng Chen, Wenhai Deng, Dandan Li, Tai Zeng, Ling Huang, Qun Wang, Jinli Wang, Weiguang Zhang, Xiaoxiao Yu, Deming Duan, Jinle Wang, Hong Xia, Hanbin Chen, Wesley Huang, Jingao Li, Dahong Zhang, Xiao-Ping Zhong, Jimin Gao

**Affiliations:** ^1^Zhejiang Provincial Key Lab for Technology and Application of Model Organisms, School of Laboratory Medicine and Life Sciences, Wenzhou Medical University, Wenzhou, China; ^2^Department of Clinical Laboratory, Ninth People's Hospital, Shanghai Jiao Tong University School of Medicine, Shanghai, China; ^3^Second Affiliated Hospital and Yuying Children's Hospital of Wenzhou Medical University Wenzhou, China; ^4^First Affiliated Hospital of Wenzhou Medical University Wenzhou, China; ^5^San Marino High School, San Marino, CA, United States; ^6^Department of Radiation Oncology, Jiangxi Cancer Hospital, Nanchang, China; ^7^Department of Urology, Zhejiang Provincial People's Hospital, Hangzhou, China; ^8^Department of Pediatrics, Division of Allergy and Immunology, Duke University Medical Center, Durham, NC, United States

**Keywords:** mucosal-associated invariant T cell, healthy Chinese cohort, phenotype, activation, age

## Abstract

Mucosal-associated invariant T (MAIT) cells, which are enriched in human blood and express a semi-invariant TCR chain, play important roles in conditions such as infectious diseases and cancer. The influence of age on levels and functional characteristics of circulating MAIT cells have not been fully addressed. Here we have collected blood samples from a large cohort of healthy Chinese individuals from newborn (cord blood) to the elderly and assessed the levels of circulating MAIT cells as well as their phenotype, activation and apoptosis status, and cytokine expression profiles after *in vitro* stimulation. We found that the frequencies of circulating MAIT cells gradually increased in blood from newborns as they progressed into adulthood (20–40 years old) but then decreased during further progression toward old age (>60 years old). The lowered numbers of circulating MAIT cells in the elderly was correlated with a gradual increase of apoptosis. A majority of circulating MAIT cells expressed the chemokine receptors CCR5 and CCR6, and most also expressed CD8 and CD45RO. Few expressed CD69 in cord blood, but the frequency increased with age. Upon *in vitro* activation with PMA plus ionomycin or IL12 plus IL18, fewer MAIT cells isolated from the young adult group expressed IFN-γ, IL17A and Granzyme B then cells from other age groups while the proportion of cells that expressed TNF-α was similar. Taken together, our data provide information for guiding the assessment of normal levels and phenotypes of MAIT cells at different ages in healthy individuals and patients.

## Introduction

Mucosal-associated invariant T (MAIT) cells are the most abundant TCRαβ^+^ T cells that recognize a single antigen in the human body. Most MAIT cells express an invariant Vα7.2-Jα33 TCRα chain (TRAV1-2-TRAJ33) paired with a limited number of TCRβ chains (1, 2). MAIT cells can recognize vitamin B2 (riboflavin) metabolites presented by the MHC class I-like molecule MR1 (1, 3, 4), which is ubiquitously transcribed (5). In addition to TCRVα7.2, circulating MAIT cells in humans also express high levels of the NK cell receptors CD161 and IL-18Rα on the cell surface (6, 7) and thus, are usually defined as TCRVα7.2^+^CD161^hi^IL18Rα^+^.

MAIT cells in both human and mice can be identified with MR1 tetramers loaded with MAIT cell ligands, such as 5-OP-RU (2, 8–10). MR1-5-OP-RU positive MAIT cells in human thymus are subdivided into three stages which are based on the expression of CD27 and CD161. The stages 1, 2, and 3 of human MAIT cells are, respectively, as follow: CD27^−^CD161^−^, CD27^+^CD161^−^, and CD27^−^CD161^+^. MAIT cells are enriched in healthy human blood, representing 1–10% of αβ T cells (6, 11, 12). MAIT cells in cord blood display a naive phenotype and are relatively low in percentages. At 2 years of age, the circulating MAIT cell number increases to an adult level and predominately manifests the CD45RO^+^ memory phenotype (6, 11, 13–15). More than 95% of the adult human blood MAIT cells are CD27^−^CD161^+^(8). Based on the expression of CD4 and CD8 on MAIT cells, MAIT cells are subdivided into cell populations of CD8^+^, CD8^−^CD4^−^, and CD4^+^ (2, 8, 16). Most of blood MAIT cells are either CD8^+^ or CD8^−^CD4^−^ cells, although a small proportion of CD8^+^CD4^+^ circulating MAIT cells has also been identified (16). In adult humans, about 80% of MAIT cells are CD8^+^ (8, 16), but this gradually decreases with age (13, 16, 17).

Growing evidence indicates that MAIT cells play important roles in immune responses against microbial infection (12, 18, 19). MAIT cells can be activated via both TCR-dependent and TCR-independent manners. Upon activation, they rapidly produce multiple cytokines such as interferon-γ (IFN-γ), interleukin-17 (IL17), and tumor necrosis factor-α (TNF-α) (7, 11). Additionally, activated MAIT cells up-regulate cytolytic products such as granzyme B and thus, have the ability to directly kill bacterial-infected target cells and tumor cells (17, 20, 21). Many studies have also revealed that MAIT cells are involved in various diseases. The levels of circulating MAIT cells are lower in patients with chronic viral infection such as in HIV and HCV (22), inflammatory bowel diseases (23–26), type 2 diabetes (10) or autoimmune diseases such as ANCA-associated vasculitis (27) and SLE (28, 29), and mucosal-associated tumors (21, 23, 30–32). However, the variations of MAIT cells in healthy humans have not been fully investigated. Although there were some published studies which mainly focused on the investigation of MAIT cells in healthy humans, these studies did not conduct an in-depth examination of the characteristics of circulating MAIT cells across a wide age range, from cord blood (CB) to elderly (13, 16, 33). Therefore, we carried out an investigation using a large cohort of healthy Chinese subjects, from new born babies to the elderly to evaluate the level of circulating MAIT cells, the composition of their subsets, the status of their phenotype, activation and apoptosis, and cytokine production profile.

## Materials and Methods

### Study Subjects

Study subjects included 379 healthy humans from newborn to 78 years old. Cord blood samples were provided by the First Affiliated Hospital of Wenzhou Medical University. Other blood samples were collected from healthy Chinese individuals during routine physical examination at the Second Affiliated Hospital and the Yuying Children's Hospital of Wenzhou Medical University. All subjects with abnormal clinical examination or history of diseases such as cancer, infectious disease and autoimmune disease were excluded from this study. Written consent was obtained from all adult participants and the parents of all non-adult participants. Blood samples were subdivided into 5 subgroups as summarized in Table 1. This study is approved (Reference Number 20160316) by the Medical Ethics Committee of Wenzhou Medical University.

**Table 1:** Demographic characteristics of the study subjects.

**Group**	**CB**	**Children (0–14 yrs)**	**Young (20–40 yrs)**	**Mid (41–60 yrs)**	**Elderly(>60 yrs)**
Female numbers	8	50	45	44	44
Age in female	0	3.72 ± 0.58	30.98 ± 0.69	49.23 ± 0.79	66.15 ± 0.94
Male numbers	5	50	45	44	44
Age in male	0	3.8 ± 0.48	29.69 ± 0.7	48.84 ± 0.79	66.52 ± 0.65
Total numbers	13	100	90	88	88
Age in total	0	3.94 ± 0.37	30.33 ± 0.49	49.03 ± 0.55	66.36 ± 0.54

*Age (years): Mean ± SEM*.

### PBMC Cell Isolation and Stimulation

PBMCs were isolated by gradient centrifugation with Ficoll-Paque Plus reagents (GE, 17-1440-02, Chicago, USA), following the manufacturer's protocol. To assess intracellular cytokine production, PBMCs were stimulated for 18 h with either 50 ng/ml PMA plus 500 ng/ml Ionomycin (eBiosecience, Massachusetts, USA) or with 10 ng/mL IL-12 (p70, PeproTech, New Jersey, USA) plus 100 ng/mL IL-18 (R&D Systems, Minneapolis, USA), with the addition of brefeldin A in the last 4 h in complete medium. Independent experiments of samples from four age groups were performed in parallel for 3–5 times, and all the data were collected. As a control, samples without stimulation were used for setting gating regions in flow cytometric analysis.

### Flow Cytometry

Staining of surface marker antigens was performed at 4°C for 15–20 min in the dark, using the following monoclonal fluorochrome conjugated antibodies: FITC-CCR6 (G034E3), FITC-CCR5 (HEK1/850), Percep5.5-TCRγδ(B1), PE-CD161 (HP-3G10), PE-Cy7, or FITC-TCRVα7.2 (3C10), Pacific Blue-CD62L (DREG-56), FITC-CD45RO (1CHL), Pacific Blue-CD69 (FN50), APC-CD127 (A019D5), APC-Cy7-CD3 (HIT3a), Pacific Blue or APC-CD8 (RPA-T8), and APC or FITC-CD4 (OKT4). All antibodies were purchased from BioLegend (San Diego, USA). Dead cells were excluded during FACS analysis. Intracellular cytokine staining was performed using fixation/permeabilization buffer (BD Biosciences, San Jose, USA), following the manufacturer's protocol. Pacific Blue-Annexin V was used for the detection of cell apoptosis immediately after isolation of PBMCs. Data was acquired using BD FACSAria^TM^ III flow cytometer and analyzed by FlowJo software (version 10.0.7; Tree Star, Inc).

### Statistical Analysis

The statistical significance was assessed using Mann–Whitney *U*-test or unpaired Student's *t*-test while the correlation analysis was performed using Spearman's test. A linear regression analysis was performed to compare the age-related changes in the level of MAIT cells and the expression of CD69 or PLZF in circulating MAIT cells. All analyses were performed using GraphPad Prism 6 (GraphPad Software, Inc.,). Data is expressed as mean ± SEM, mean values are shown with horizontal bars, and *p* < 0.05 are considered as statistically significant (^*^p < 0.05, ^**^p < 0.01, ^***^p < 0.001, ^****^p < 0.0001).

## Results

### Increased Circulating MAIT Cell Frequency From CB to Young Subjects, but Decreased From Young to Elderly Subjects

**Figure 1:**
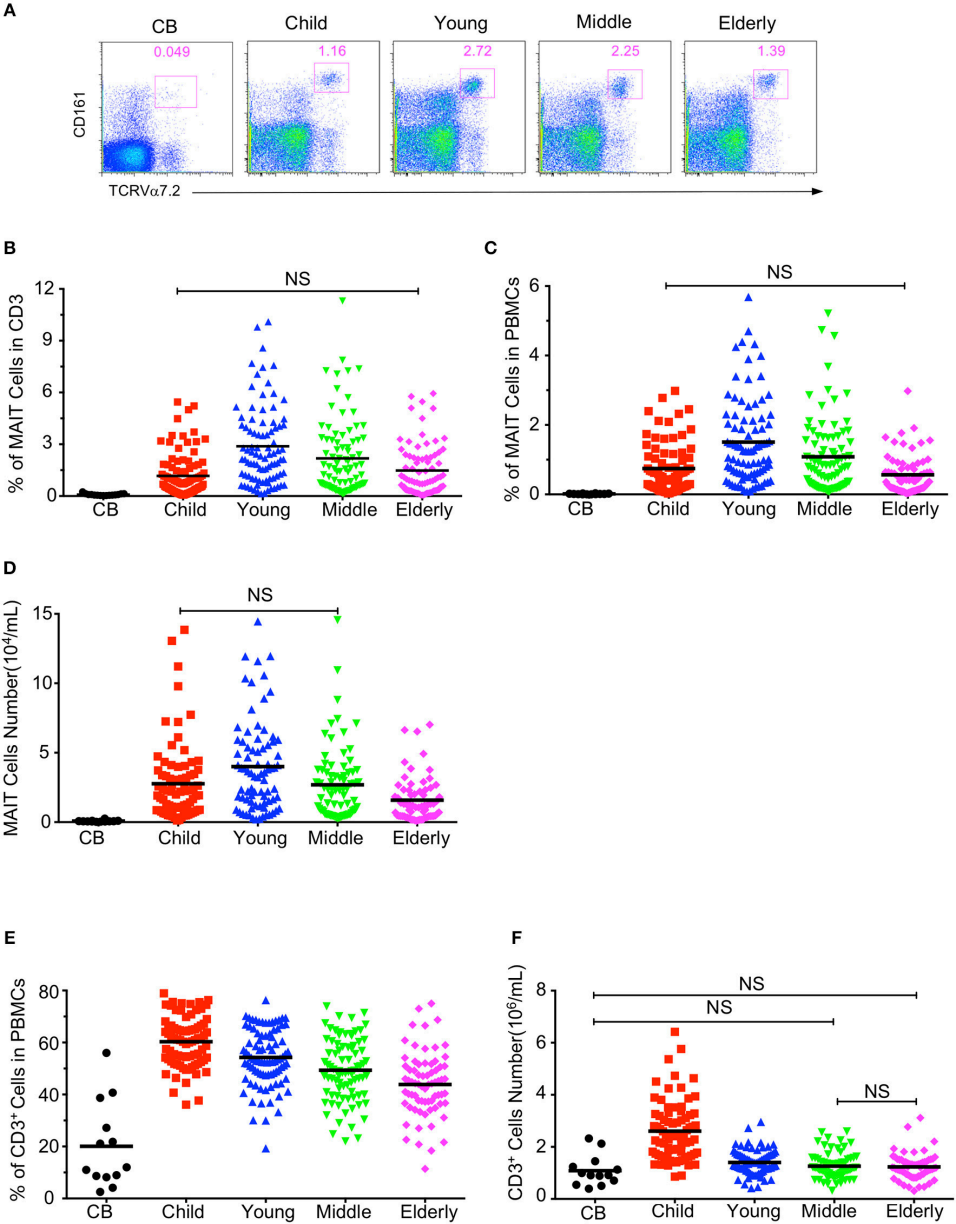
Circulating MAIT cell and CD3^+^ T cell frequencies and numbers in different cohorts. Freshly isolated PBMCs from 379 healthy individuals (grouped as shown in Table 1) were analyzed by flow cytometry. MAIT cells were gated as 7-AAD-TCRγδ^−^ CD3^+^TCRVα7.2^+^CD161^hi^. **(A)** Representative FACS plots showing TCRVα7.2 and CD161 expression in live gated TCRγδ^−^CD3^+^ cells. Numbers adjacent to the rectangles are percentages within live gated TCRγδ^−^CD3^+^ cells. **(B)** MAIT cell percentages in CD3^+^ TCRγδ^−^ T cells. **(C)** MAIT cell percentages in viable PBMCs. **(D)** Absolute MAIT cell numbers in PBMCs per milliliter of blood. **(E)** CD3^+^ cell percentages in viable PBMCs. **(F)** CD3^+^ cells absolute Number. Each symbol represents an individual subject. Statistical significance was assessed using the Mann-Whitney *U*-test. Horizontal bars show the mean values, *p* < 0.05 were considered as statistically significant, unless otherwise indicated as NS (NS, Not significant).

Firstly, we defined human blood circulating MAIT cells as CD3^+^TCRVα7.2^+^TCRγδ^−^CD161^hi^ cells by flow cytometry (Supplemental Figure 1) as suggested by a previous report (8). To determine how age may influence the frequency of circulating MAIT cells in humans, we examined MAIT cells in blood samples from 379 healthy individuals, which included 13 cord blood, 100 children (under 14 years old), 90 youths (20–40 years old), 88 middle-age persons (41–60 years old), 88 elderly (above 60 years old) (Table 1). The frequencies of Vα7.2^+^CD161^hi^ MAIT cells in the CD3^+^TCRγδ^−^ population progressively increased when comparison is made from groups of CB to youth, at a respective average frequency of 0.09, 1.17, and 2.88% in the CB, Children and Youth groups. However, MAIT cell frequencies progressively decreased from groups of youth to elderly, at a respective average frequency of 2.88, 2.18, and 1.42% in the youth, middle-age, and elderly groups (Figures 1A,B). A similar trend was observed in the MAIT cell frequencies as relative to whole PBMCs (CB, mean ± SEM: 0.01 ± 0.003%; Children, 0.75 ± 0.08%; Youth, 1.51 ± 0.13%; Middle-age, 1.09 ± 0.12%; and Elderly, 0.56 ± 0.07%) (Figure 1C). Corresponding to the changes in frequency, the numbers of MAIT cells increased from CB to youth, and then decreased from youth to elderly (CB, 0.076 ± 0.017; Children, 2.78 ± 0.31; Youth, 3.92 ± 0.34; Middle-age, 2.6 ± 0.29; and Elderly, 1.53 ± 0.19 × 10^4^/ml) (Figure 1D). Therefore, both the percentage and number of MAIT cells are very low in cord blood, increase during childhood, peak during youth, and then progressively decreased from middle to old age.

To determine whether age-associated changes in MAIT cells were due to similar changes in CD3^+^ T cells, we analyzed the CD3^+^ cells in PBMCs. As shown in Figures 1E,F, the percentage and number of CD3^+^ cells were the lowest in cord blood (CB, 20.07 ± 4.55%), the highest in the children group (60.32 ± 1.08%), and gradually decreased from youth to elderly (Youth, 54.26 ± 1.16%; Middle-age, 49.34 ± 1.3%; Elderly, 43.85 ± 1.47%).

### Impacts of Age and Gender on the Level of Circulating MAIT Cells

**Figure 2:**
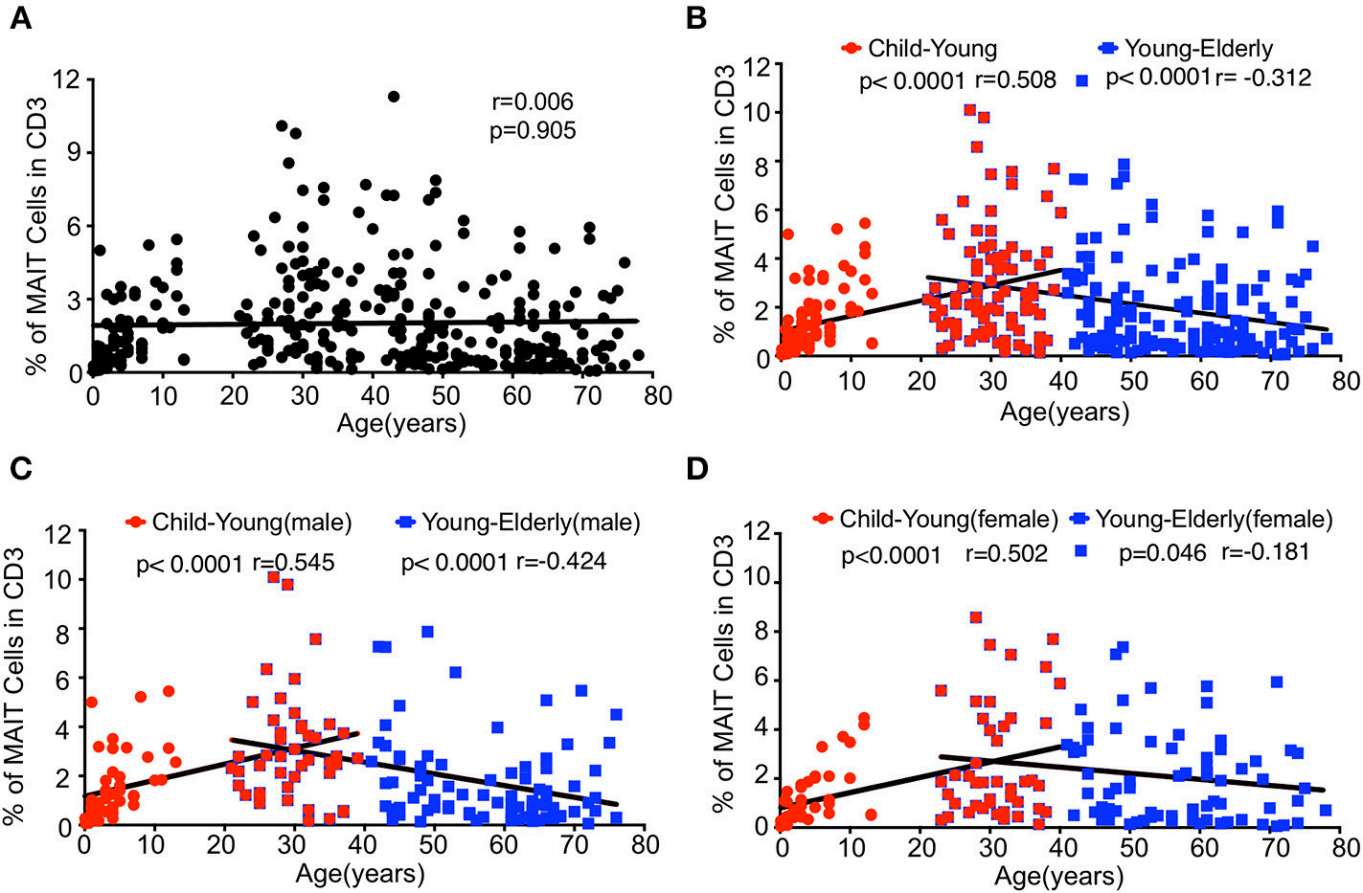
Relationship between age and MAIT cell frequencies in healthy subjects. Data from the same cohort of healthy individuals (CB is excluded) in Figure 1 were analyzed for Spearman's correlation between age and MAIT cell frequencies. **(A)** The correlation between age and MAIT cell percentages in CD3^+^ cells of the entire cohort. **(B)** The correlation between age and MAIT cell percentages in CD3^+^ cells of the entire cohort but separated into Child to Young and Young to Elderly groups. **(C,D)** The correlation between age and MAIT cell percentages in CD3^+^ cells of the male **(C)** and female **(D)** cohort separated into Child to Young and Young to Elderly groups. Correlation analysis was performed using Spearman's test. Linear regression analysis was performed to compare the age-related changes in MAIT cell levels **(A–D)**.

Although it has been reported that there is an inverse correlation between age and frequency of the circulating MAIT cell (13, 17), our data shown in Figure 1 suggest that the relationship between age and frequencies of circulating MAIT cells cannot be correlated so simply. Indeed, the Spearman's correlation coefficient between age and the percentage of MAIT cells in CD3^+^TCRγδ^−^ T cells relative to the entire cohort did not reflect any relationship between age and the percentage of MAIT cells (*r* = 0.006, *p* = 0.905) (Figure 2A). However, when the entire cohort was separated into two age groups, child to youth and youth to elderly, a positive correlation between age and the frequency of MAIT cells (*r* = 0.508, *P* < 0.0001,) was observed from child to youth while a negative correlation between age and the frequency of MAIT cell (*r* = −0.312, *p* < 0.0001) (Figure 2B) was shown from youth to elderly. To determine if such correlations existed in terms of gender, the cohort was categorized into male and female, and then, a similar analysis was performed of each group. Accordingly, a similar positive correlation was observed from child to youth, and a negative correlation from youth to elderly in both gender groups (Figures 2C,D); although, a weaker inverse correlation was shown in the female category from youth to elderly. No significant differences were found between female and male in frequency of the circulating MAIT cell (Supplemental Figure 2).

### Phenotype of Circulating MAIT Cells in Healthy Subjects

**Figure 3:**
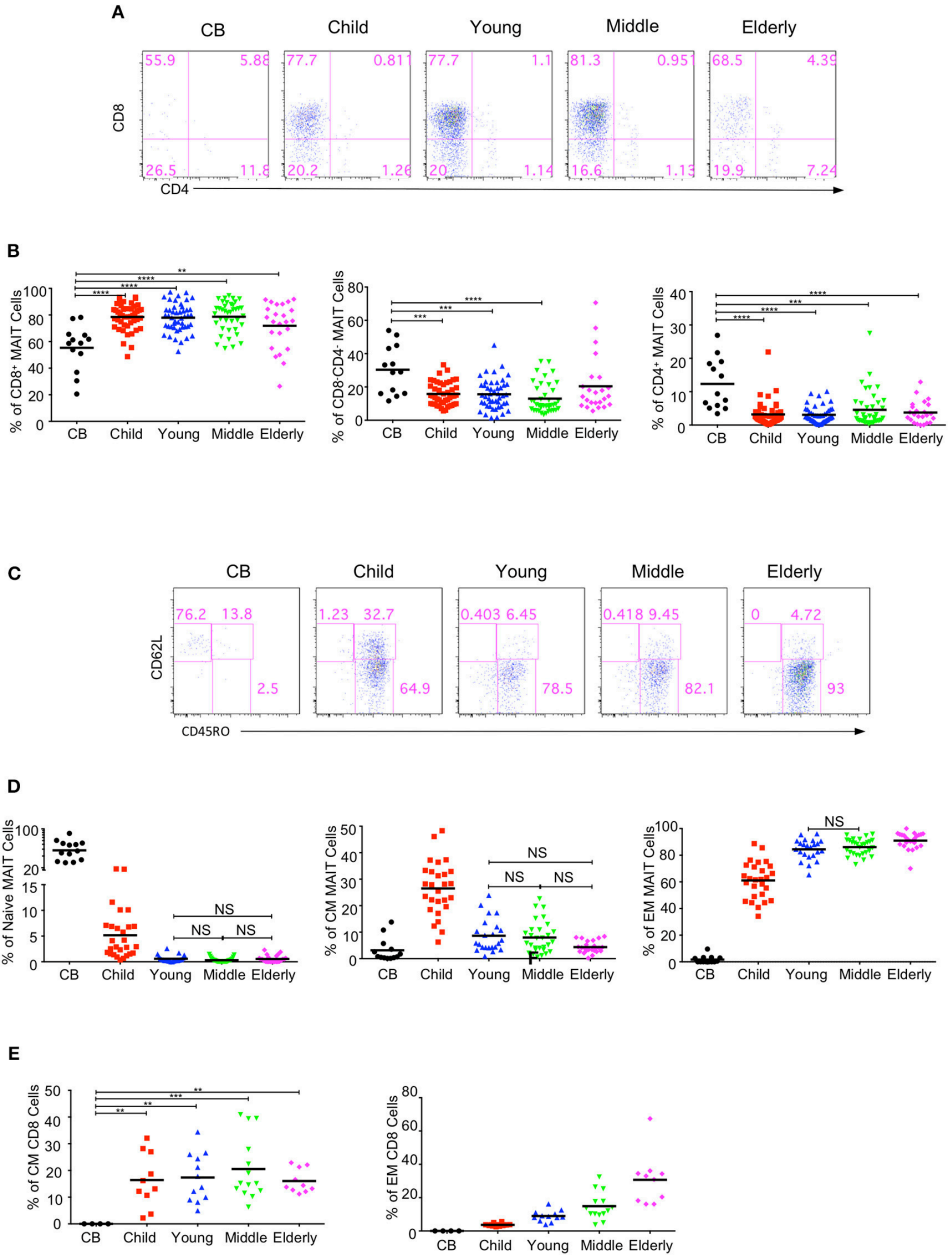
MAIT cells subsets and effector phenotypes in different age groups. MAIT cells were analyzed for CD4 and CD8 or CD62L and CD45RO expression. **(A)** CD4 and CD8 expression in MAIT cells. **(B)** The percentages of CD8^+^CD4^−^ (left), CD4^−^CD8^−^ (middle), and CD4^+^CD8^−^ (right) MAIT cell subsets In **(A,B)**, CB (*n* = 13), Child (*n* = 44), Young (*n* = 45), Middle (*n* = 38), Elderly (*n* = 24). Statistical significance was assessed using the Mann-Whitney *U*-test. Horizontal bars show the mean values, *p* < 0.05 were considered as statistically significant. (^**^p < 0.01, ^***^p < 0.001, ^****^p < 0.0001). **(C)** CD45RO and CD62L expression in MAIT cells. **(D)** Percentages of naive (CD45RO^−^CD62L^+^, left), CM (CD45RO^+^CD62L^+^, middle), and EM (CD45RO^+^CD62L^−^, right) MAIT cells In **(C,D)**, CB (*n* = 13), Child (*n* = 27), Young (*n* = 23), Middle (*n* = 29), Elderly (*n* = 24). Statistical significance was assessed using the Mann-Whitney *U* test. Horizontal bars show the mean values, *p* < 0.05 were considered as statistically significant, unless otherwise indicated as NS (NS, Not significant). **(E)** Percentages of CM (left), and EM (right) regular CD8^+^ cells. Each dot represents an individual subject. Statistical significance was assessed using the Mann-Whitney *U* test. Horizontal bars show the mean values, *p* < 0.05 were considered as statistically significant. Statistically significant differences were found between all the groups in the right plot of **(E)** (^**^p < 0.01, ^***^p < 0.001, ^****^p <0.0001).

MAIT cells are subdivided into CD4^+^, CD8^+^ and double negative (DN) subsets (2, 8, 16). We analyzed the expression of CD4 and CD8 in MAIT cells in the same cohort of healthy individuals. CD4^−^CD8^+^ MAIT cells accounted for about 50% (55.32% ± 4.72) in the CB group, 58–80% in the children group (78.51 ± 1.423%), 75% in the youth group (77.97 ± 1.54%), and 75 % in the middle-age group (78.66 ± 1.82%), but decreased to about 70% in the elderly group (71.86 ± 3.56%). CD4^+^CD8^−^ MAIT cells were rare, accounting for about 10% in the CB but <10% in the other age groups. On the other hand, CD4^−^CD8^−^ DN MAIT cells accounted for about 30% (30.32 ± 4.04%) of the MAIT cells in the CB group. In the children, young, middle-age and elderly groups, the percentages of DN MAIT cells were less than in the CB group, reflecting a lower percentage than CD8^+^ cells but a higher percentage than CD4^+^ MAIT cells, accounting for 15.78 ± 1.03, 15.61 ± 1.31, 12.96 ± 1.4, and 20.04 ± 3.96%, respectively (Figures 3A,B). Of note, there is a considerable variation for each subset of MAIT cells among individuals across all the age groups.

In peripheral blood, MAIT cells quickly acquire a memory phenotype several months after birth (6, 11, 13). MAIT cells are defined into naive (CD62L^+^CD45RO^−^), central memory (CM, CD62L^+^CD45RO^+^), and effect memory (EM, CD62L^−^CD45RO^+^) populations. MAIT cells in CB were predominantly naive (55.82% ± 5.42), with very low percentages of CM (3.15% ± 1.23) and EM (1.69% ± 0.76) populations. Naive MAIT cells rapidly differentiated into EM and CM cells in the children group and were thus very rare in the youth, middle-age and elderly groups (Figures 3C,D). In the children group, the EM population was predominant, while the naive population became the minority. Also, the CM population showed an obvious increase as compared with that of the CB group. Along with the age increase from children to elderly, the EM population increased while the CM population decreased. However, the CM cells in regular CD8^+^ T cells were found to be comparable among the four groups (children, youth, middle-age, and elderly); i.e., about 15% regular CD8^+^ T cells were CM cells (Figure 3E). Meanwhile, the EM cells of regular CD8^+^ T cells in each group gradually increased from CB to elderly with a respective average frequency of 0, 3.67, 8.93, 14.89, and 30.70% in the CB, children, youth, middle-age, and elderly groups (Figure 3E). Of note, the children group exhibited the widest variations, which was consistent with previous observations that MAIT cells phenotypically evolved quickly within the first few years of life after birth (11).

### Expressions of Circulating MAIT Cell-Related Biomarkers in Different Age Groups

**Figure 4:**
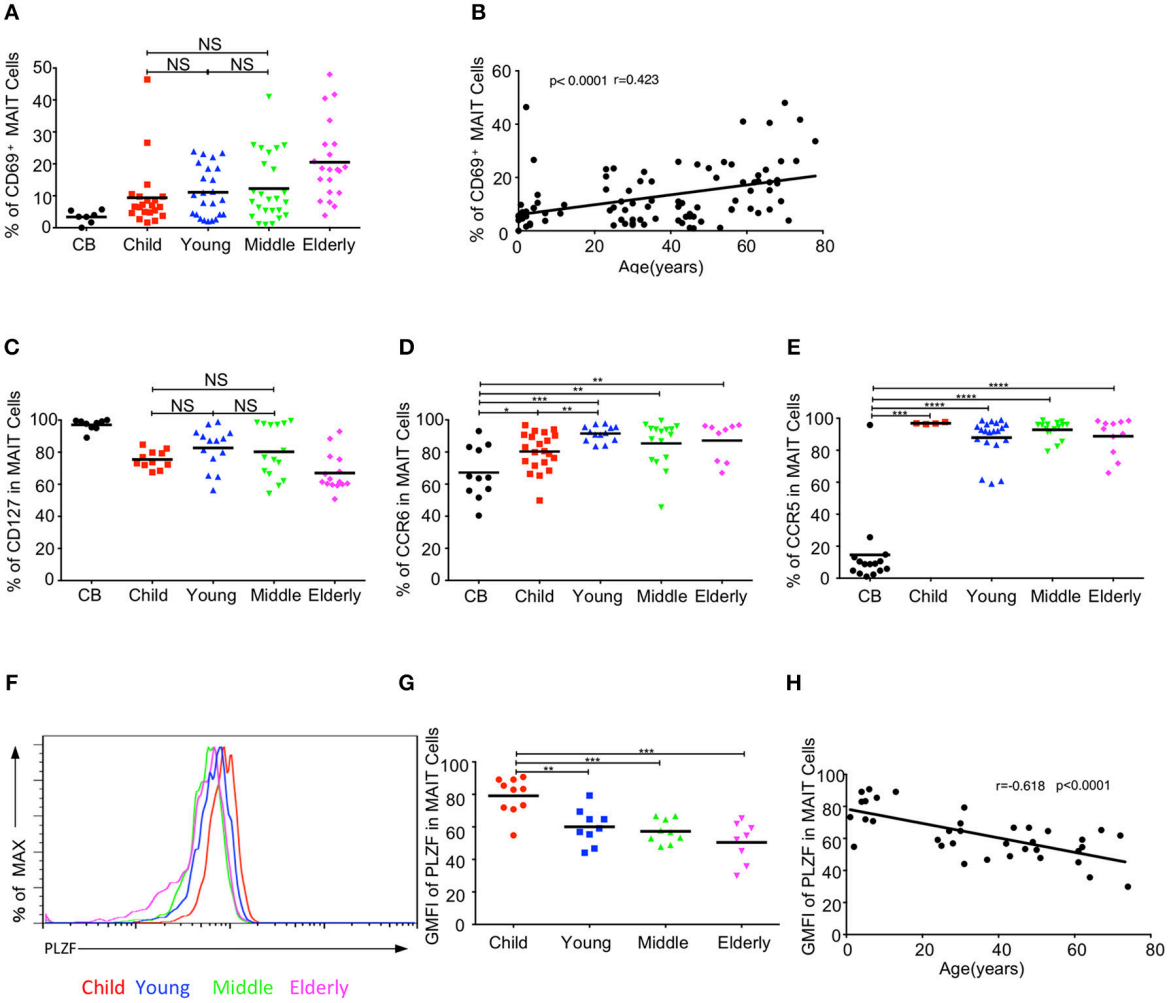
MAIT cell-related biomarker expression levels. **(A)** CD69 expression in MAIT cells. **(B)** Correlation between age and CD69 expression on circulating MAIT cells. **(C)** CD127 expression in MAIT cells. **(D,E)** Expression CCR6 **(D)** and CCR5 **(E)** on MAIT cells. **(F)** Representative of FACS histograms showing PLZF expression in MAIT cells from Child (red line), Young (blue line), Middle (green line), and Elderly (pink line). Data representative of at least 3 donors. **(G)**The gMFI of PLZF in MAIT cells. **(H)** Correlation between age and GMFI of PLZF in circulating MAIT cells. Each dot represents an individual donor. **(A,C)** Statistical significance was assessed using the Mann-Whitney *U*-test. Horizontal bars show the mean values, *p* < 0.05 were considered as statistically significant, unless otherwise indicated as NS (NS, Not significant). **(D,E,G)** *p* < 0.05 were considered as statistically significant (^*^p < 0.05, ^**^p < 0.01, ^***^p < 0.001, ^****^p < 0.0001). **(B,H)** Correlation analysis was performed using Spearman's test. Linear regression analysis was performed to compare the age-related changes in CD69/PLZF expression level.

We further examined whether the activation status of MAIT cells varied among the different age groups in the cohort. The expression of the T cell activation marker, CD69, gradually increased from CB to elderly. The respective average frequencies of CD69^+^ MAIT cells in the CB, children, young, middle-age, and elderly groups were 3.36, 9.39, 11.1, 12.25, and 20.5% (Figure 4A). There was a positive correlation between age and the frequency of CD69^+^ MAIT cells (*r* = 0.423, *p* < 0.0001) (Figure 4B).IL7 receptor signal can license liver intrasinusoidal MAIT cell activation and prime MAIT cells for IL17 expression (34, 35). Consistent with a previous study (11), we found that blood MAIT cells expressed a high level of CD127 (IL7Rα)^+^ in the entire cohort. The percentage of CD127 in circulating MAIT cells was the highest in the CB group (97.04% ± 1.15) and the lowest in the elderly group (67.02% ± 3) (Figure 4C). MAIT cells expressed the homing receptors CCR6 and CCR5, which enabled them to migrate to the liver, intestines and lungs (11, 12, 36). The frequency of CCR6^+^ MAIT cells gradually increased from CB to young, with the respective average frequencies of 67.21, 80.30, and 91.54% in the CB, children, and youth age groups. Although the frequencies of CCR6^+^ MAIT cells appeared lower in the middle-age (85.35%) and elderly (87.16%) groups than that in the youth group, the differences among the three groups were not statistically significant (Figure 4D). Newborns, with the exception of one CB, exhibited a low frequency of CCR5^+^ MAIT cells (14.56% ± 6.01). However, more than 85% MAIT cells expressed CCR5 in the other groups (Figure 4E). PLZF, a signature transcription factor of the innate-like lymphocyte, is critical for the maturation of MAIT cells in the transition of stage 2 to stage 3, within thymus and acquisition of effector function (8). The geometrical mean of the fluorescence intensity (GMFI) of PLZF in the circulating MAIT cells was the highest in the CB group and then gradually decreased with age (Figures 4F,G). An analysis of the relationship revealed a strong correlation between age and the decrease of GMFI in PLZF (*P* < 0.0001, *r* = −0.618) (Figure 4H).

### Increased MAIT Cells Apoptosis in the Elderly

**Figure 5:**
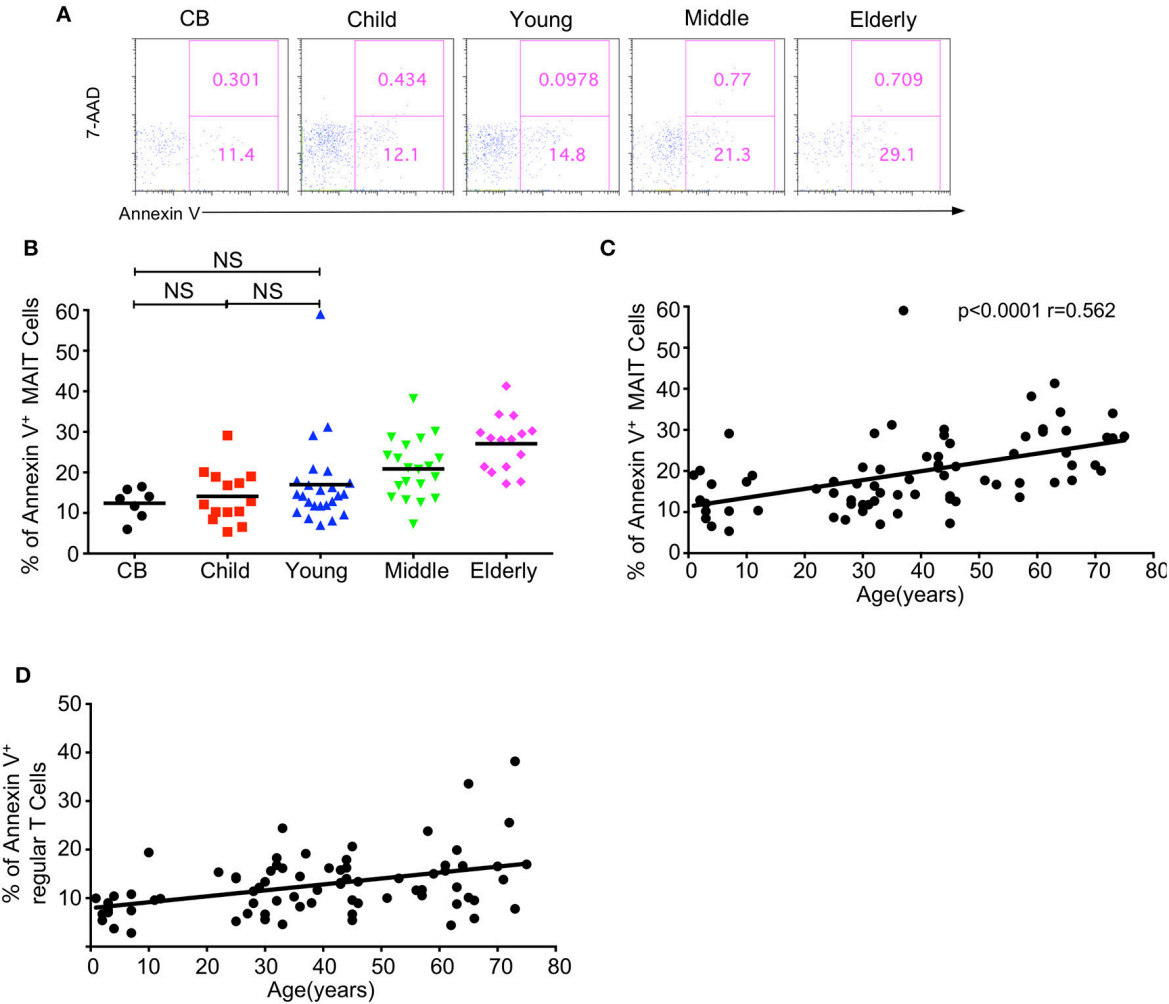
Apoptosis of MAIT cells in healthy humans. Freshly isolated PBMCs from 7 CB, 14 Child, 24 Young, 20 Middle and 15 Elderly healthy humans were stained with CD3, CD161, TCRVα7.2, and TCRγδ, and then stained with 7-AAD and Annexin V. **(A)** Representative of FACS plots showing 7AAD and Annexin V staining after gating on MAIT cells in the indicated groups. Numbers in outlined rectangles were percentages of 7-AAD^+^Annexin V^+^(top panel) or 7-AAD^−^Annexin V^+^ (bottom panel) cells. **(B)** Percentages of Annexin V^+^ cells in the indicated groups; Statistical significance was assessed using the Mann-Whitney *U*-test. Horizontal bars show the mean values, *p* < 0.05 were considered as statistically significant, unless otherwise indicated as NS. (NS, Not significant). **(C)** The correlation between age and Annexin V^+^ cell percentages in MAIT cells of the entire cohort (CB is excluded). **(D)** The correlation between age and Annexin V^+^ cell percentages in regular cells of the entire cohort (CB is excluded). Correlation analysis was performed using Spearman's test. Each dot represents an individual donor **(B–D)**.

Given the decreased level of circulating MAIT cells with age, we sought to determine if the apoptosis of MAIT cells varied among different age groups by staining them with 7-AAD and Annexin V. The percentages of Annexin V positive apoptotic MAIT cells gradually increased from CB to elderly with the respective average frequencies of 12.58, 14.10, 16.98, 20.88, and 27.06% in the CB, children, young, middle-age, and elderly groups (Figures 5A,B). There was a positive correlation between age and the percentage of Annexin V^+^ cells in MAIT cells (*p* < 0.0001, *r* = 0.562) (Figure 5C). Meanwhile, the same phenotype was found in regular T cells, i.e., increased apoptosis T cells with age (Figure 5D). These data indicated that increased cell death might contribute to the decrease of MAIT cells from youth to elderly.

### Cytokine Secretion of MAIT Cells Upon Activation in Healthy Subjects

**Figure 6:**
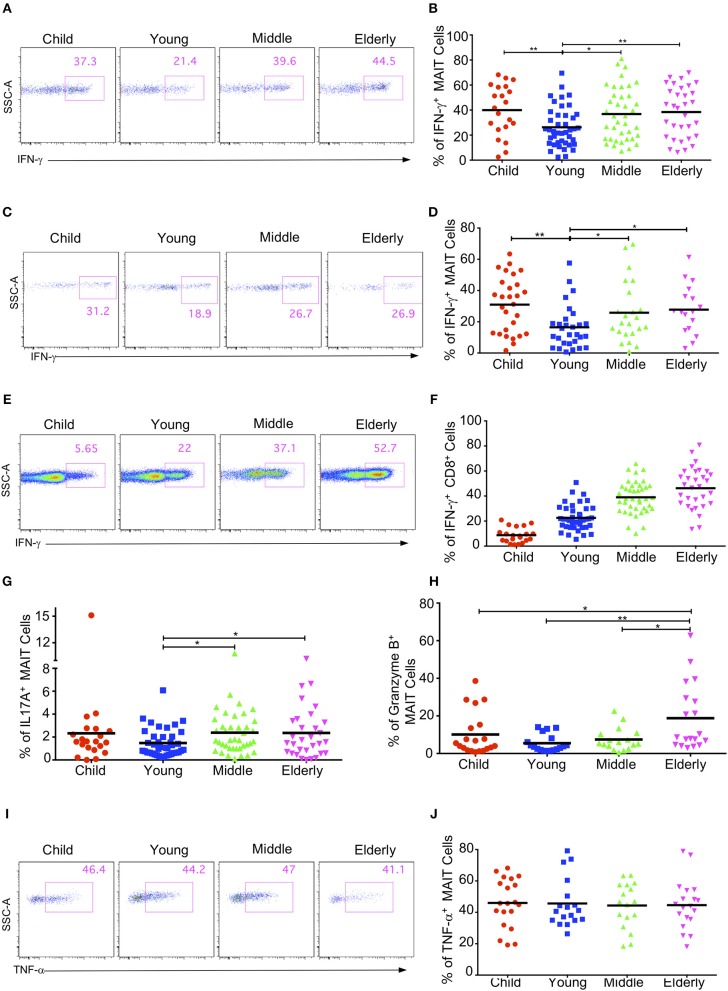
Cytokine production by circulating MAIT cells upon activation within different cohorts. Freshly isolated PBMCs were stimulated with either PMA plus ionomycin for 4h **(A,B, E–J)** or stimulated with IL-12 plus IL-18 for 20 h **(C,D)**, with the addition of brefeldin A in the last 4h. After stimulation, harvested cells were stained with MAIT cells surface marker CD3, CD161 and TCRVα7.2, followed by intracellular staining for IFN-γ, IL17A, Granzyme B, and TNF-α. LIVE/DEAD staining was used to exclude dead cells. **(A,C,E)** Representative FACS plots showing IFN-γ expression in MAIT cells after PMA plus ionomycin stimulation **(A)**, after IL-12 plus IL18 stimulation **(C)**, or in non-MAIT CD8^+^ cells after PMA plus ionomycin **(E)**. **(B,D,F)** Percentages of IFN-γ producing cells in MAIT cells after PMA plus ionomycin stimulation **(B)**, after IL-12 plus IL18 stimulation **(D)**, or in non-MAIT CD8^+^ cells after PMA plus Ionomycin **(F)**. **(G)** Percentages of IL17A producing cells in MAIT cells. **(H)** Percentages of Granzyme B producing cells in MAIT cells. **(I)** Representative FACS plots showing percentages of TNF-α producing cells in MAIT cells. **(J)** Percentages of TNF-α producing cells in MAIT cells. Statistical significance was assessed using unpaired Student's *t*-test; Each dot represents an individual donor; Horizontal bars show the mean values, *p* < 0.05 were considered as statistically significant. Statistically significant differences were found between all the groups in **(F)**. (^*^p < 0.05, ^**^p < 0.01).

MAIT cells have the capacity to secrete cytokines such as IFN-γ, IL17A, and TNF-α, and to express granzyme B after stimulation (11, 18, 37, 38). We next investigated the cytokine production of circulating MAIT cells in healthy subjects after an *in-vitro* stimulation with PMA and ionomycin. We didn't detect any cytokine secretion of MAIT cells in the CB group with the naive phenotype (data not shown), so we compared the cytokine production in the children, youth, middle-age, and elderly groups. As the percentage of EM cells in MAIT cells gradually increased with age, we speculated that the cytokine secretion of MAIT cells might also gradually increase with age. Surprisingly, MAIT cells in the youth group had the lowest IFN-γ^+^ cells (26.24% ± 2.44%), with other groups being not obviously different (Children: 39.95 ± 4.41%; Middle-age: 36.83 ± 3.36%; Elderly: 38.41 ± 3.54%) (Figures 6A,B). The same pattern was also observed when MAIT cells were stimulated with IL12 and IL18 (Figures 6C,D). Different from MAIT cells, the expression of IFN-γ in conventional CD8^+^ T cells gradually increased from children to elderly (Children, 8.74% ± 1.3, Youth, 22.55% ± 1.54, Middle-age, 38.99% ± 2.04, Elderly, 46.26% ± 2.94) (Figures 6E,F). These observations highlighted the differences between MAIT cells and conventional CD8^+^ T cells.

We further measured the expression of IL17A, granzyme B, and TNF-α in the MAIT cells from different age groups and found that the percentages of IL17A-expressing cells in the circulating MAIT cells of healthy subjects were very low (around 1%−3%). Similar to IFN-γ, MATI cells had the lowest percentage of IL17A-expressing cells in the youth group, while the other three groups had similar percentages of IL17A-expressing cells (Figure 6G). The MAIT cells in the youth group also had the lowest percentage of granzyme B expression (5% ± 1.08), whereas the children and the middle-age groups had similar percentages of granzyme B expression (10.1% ± 2.56 and 7.5% ± 1.46). Meanwhile, the highest percentage of granzyme B expression came from the elderly group (18.77% ± 4.06) (Figure 6H). However, the percentage of TNF-α expression appeared unaffected by age, and about 40% MAIT cells produced TNF-α after PMA and ionomycin stimulation (Figures 6I,J). In general, the MAIT cells in the youth group appeared to exhibit weak effector functions as compared with those in the other age groups. This indicates that there might be some specific environmental factors in young subjects that suppress MAIT cell effector function.

## Discussion

MAIT cells belong to innate-like T lymphocytes. Until now, our understanding of peripheral blood MAIT cells in healthy individuals at different ages is still incomplete. Khaitan's group found that there is no significant correlation between the frequency of CD8^+^ MAIT cells and age in healthy children (30). Booth's group assessed the MAIT cells subsets (CD8^+^ and DN) from children to elderly (17). These studies, as well as those from Dusseaux's group (11), were suffered by small sample sizes. Although Lee's group had examined MAIT cells in a large cohort of healthy Korean subjects from youth to elderly (16), no data on CBs and children were presented. Here, we conducted a comprehensive investigation on the levels, subsets, phenotypic characteristics, homing proteins, activation and apoptosis status, and cytokine expression in a relatively large cohort of healthy subjects from CB to elderly. We have made the following observations: (1) peripheral blood MAIT cells were the lowest in CBs, which then progressively increased until youth, and then decreased from youth to elderly, correlating with a gradual increase in apoptosis; (2) circulating MAIT cells expressed the chemokine receptors CCR5 and CCR6, both of which were dynamically regulated; (3) the majority of circulating MAIT cells were CD8^+^ and displayed a CD45RO^+^ memory phenotype, but a small proportion of circulating MAIT cells were CD69^+^, which increased overtime; (4) upon activation with different stimuli, circulating MAIT cells expressed multiple cytokines that were also deferentially regulated along with ages, showing the lowest expression in youth.

Since MR1 tetramers for MAIT cell detection were not available when carrying out the study, we adopted a surrogate marker combination approach where MAIT cells are typically defined as CD3^+^TCRVα7.2^+^CD161^hi^ cells, which is largely coincident with the MR1 tetramer^+^ population in human according to the pioneer study on MR1 tetramer(2) and another study(10). It has to be noted that, only 2–15% cord blood CD3^+^TCRVα7.2^+^CD161^hi^ cells are MR1 tetramer^+^, even though the percentages of MR1 tetramer^+^ cells increase rapidly to become the vast majority population within CD3^+^TCRVα7.2^+^CD161^hi^ cells by 6 months of age in human (14). Additionally, 20% of MR1-tetramer^+^ MAIT cells lack CD161 expression (8). Changes in CD161^−^ MAIT cells among different age groups may not be revealed in our study. Future studies with MR1 tetramers will be required to extend present study on cord blood MAIT cells and age-associated changes of the CD161^−^ population of MAIT cells.

MAIT cells expressed the homing receptors CCR5 and CCR6, which enable them to migrate to the liver, intestines and lungs (11, 12, 36), but no study has ever reported on the age-related changes of these homing proteins on circulating MAIT cells. Thus, we first provided the data to address this issue, and then found a strong association between age and CCR5 and CCR6 expression. From CB to youth, CCR6 on MAIT cells gradually increased and maintained at similarly high levels afterwards. CCR5 expression on MAIT cells was low in CBs, with an average frequency of 14.56%. However, it peaked in children, suggesting a quick acquisition of CCR5 expression in MAIT cells.

We have demonstrated that MAIT cells increased in both percentage and number from CB to youth, and then gradually decreased from youth to elderly, in both female and male subjects. Because these trends did not correlate with those of conventional T cells, these data suggested specific age-related properties of peripheral blood MAIT cells that might be distinctive from conventional T cells. Since the apoptosis of MAIT cells gradually increased from CB to elderly, there was a possibility that this increase in apoptosis is contributive toward the decrease in MAIT cells from youth to elderly. Interestingly, a study performed on 100 Korean children to elderly showed a negative correlation between age and the percentage of MAIT cells or an absolute number with MAIT cells peaking in their third and fourth decenniums (13). At present, we cannot determine whether the differences between healthy Chinese and Korean subjects resulted from racial and/or environmental factors.

We found that the expression of CD69, a T cell activation protein, on MAIT cells gradually increased from CB to elderly, indicating that more MAIT cells were activated in older populations than in younger ones.

Upon activation, MAIT cells can produce several cytokines (11, 18, 37, 38). To the best of our knowledge, we are the first to assess IFN-γ, TNF-α, IL17A, and granzyme B levels in blood MAIT cells across a wide range of ages. We found that IFN-γ-producing MAIT cells were the lowest in the youth group but similar among the children, middle-age and elderly groups after stimulation with PMA plus ionomycin. Interestingly, two previous reports had found comparable IFN-γ levels between the youth and elderly groups (21, 33). Such differences could result from differences in sample size, race and other factors. Since MAIT cells have been shown to respond to bacteria infected antigen-presenting cells by producing IFN-γ through the cognate interaction between MR1 and the TCR [*E.coli* (19), *H.pylori* (17), *Mycobacterium tuberculosis* (39), and pulmonary Legionella longbeachae(40)], it would be interesting to examine the cytokine production in MAIT cells from different age groups of population, under the physiologic stimulation via MR1 using a model of THP1 cell lines that were pre-exposed to paraformaldehyde-fixed *Escherichia coli*. Moreover, MAIT cells express a high level of IL18 receptor (IL-18R), which may equip these cells for rapid activation by IL18. Indeed, our data showed that IL12 plus IL18 could potently activate MAIT cells to produce IFN-γ.

Put together, our data demonstrated that circulating MAIT cells in healthy subjects from CB to elderly are regulated by intrinsic properties of MAIT cells, such as apoptosis, and by environmental factors, such as microbiota. Functionally, MAIT cells in youths produce lower levels of IFN-γ, IL17A, and granzyme B. Therefore, our findings provide important information about circulating MAIT cells, which is helpful to the understanding of MAIT cell changes in healthy individuals and patients in a variety of clinical settings.

## Author Contributions

PC, WD, DZ, JL, and X-PZ designed the study. JinleW, HX, and HC recruited the study participants. PC, DL, TZ, LH, QW, JinliW, WZ, XY, DD, and WH performed the experiments and acquired data. PC, WD, and DL were involved in data analysis and interpretation. PC and WD wrote the manuscript. X-PZ and JG revised the manuscript. All authors approved the final version of the manuscript.

### Conflict of Interest Statement

The authors declare that the research was conducted in the absence of any commercial or financial relationships that could be construed as a potential conflict of interest.

## Supplementary Material

Supplemental Figure 1 The MAIT cells gating strategy. Freshly isolated PBMCs from healthy individuals were analyzed by flow cytometry. MAIT cells were gated as 7-AAD^−^TCRγδ^−^CD3^+^TCRVα7.2^+^CD161^hi^.

Supplemental Figure 2 Distribution of MAIT cell frequencies in Female (F) and Male (M) of entire groups. Freshly isolated PBMCs from 379 healthy individuals (grouped as shown in Table 1) were analyzed by flow cytometry. Each symbol represents an individual subject. Statistical significance was assessed using the Mann-Whitney *U*-test. Horizontal bars show the mean values, *p* < 0.05 were considered as statistically significant.
