# Supplementary figures and images for: Circulating Mucosal-Associated Invariant T Cells in a Large Cohort of Healthy Chinese Individuals From Newborn to Elderly
Source: Front Immunol. 2019 Feb 19;10:260. doi: 10.3389/fimmu.2019.00260 (PMC6389679; doi:10.3389/fimmu.2019.00260)

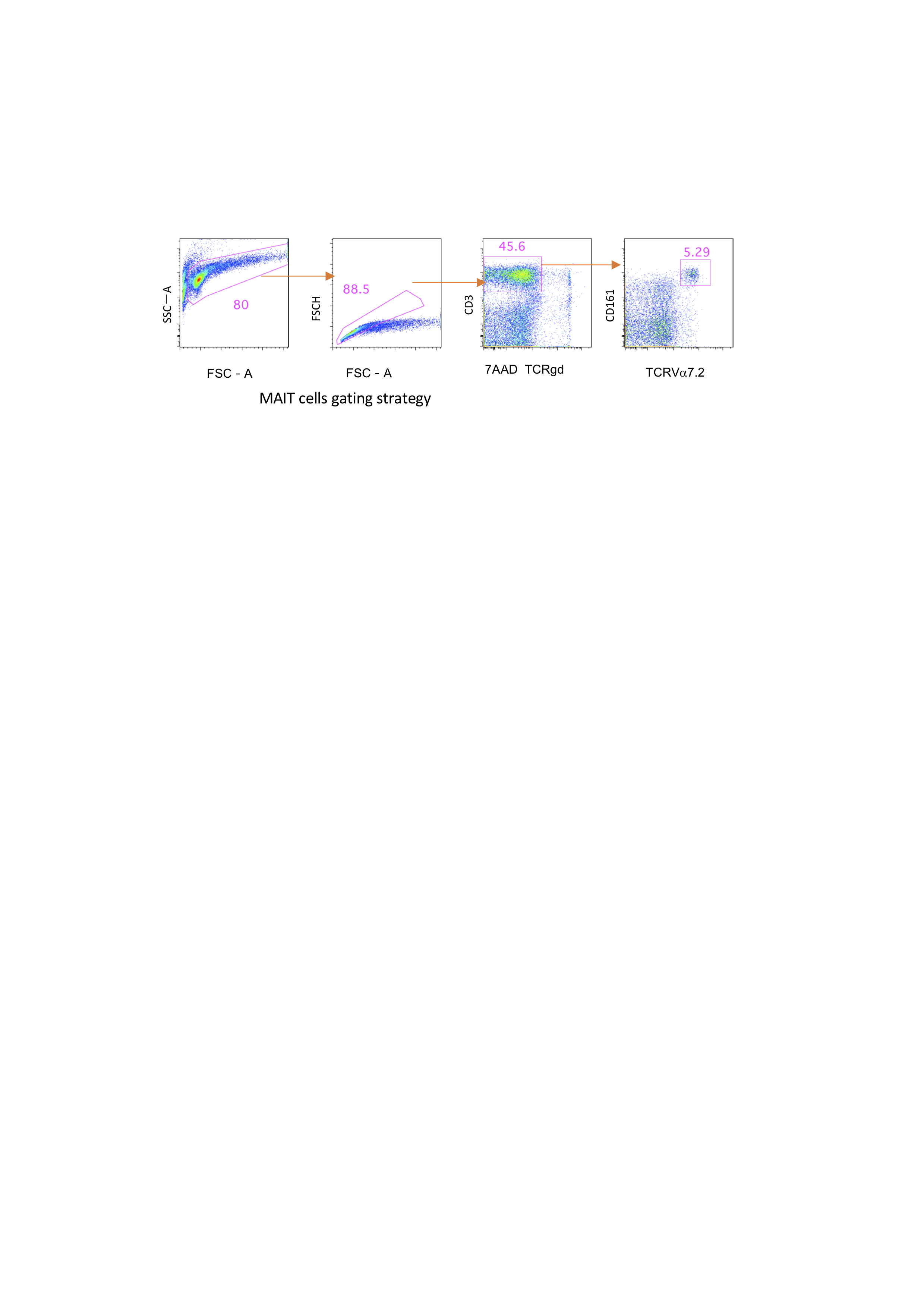

Supplement: Supplemental Figure 1 — The MAIT cells gating strategy. Freshly isolated PBMCs from healthy individuals were analyzed by flow cytometry. MAIT cells were gated as 7-AAD−TCRγδ−CD3+TCRVα7.2+CD161hi. [file Image_1.tif]

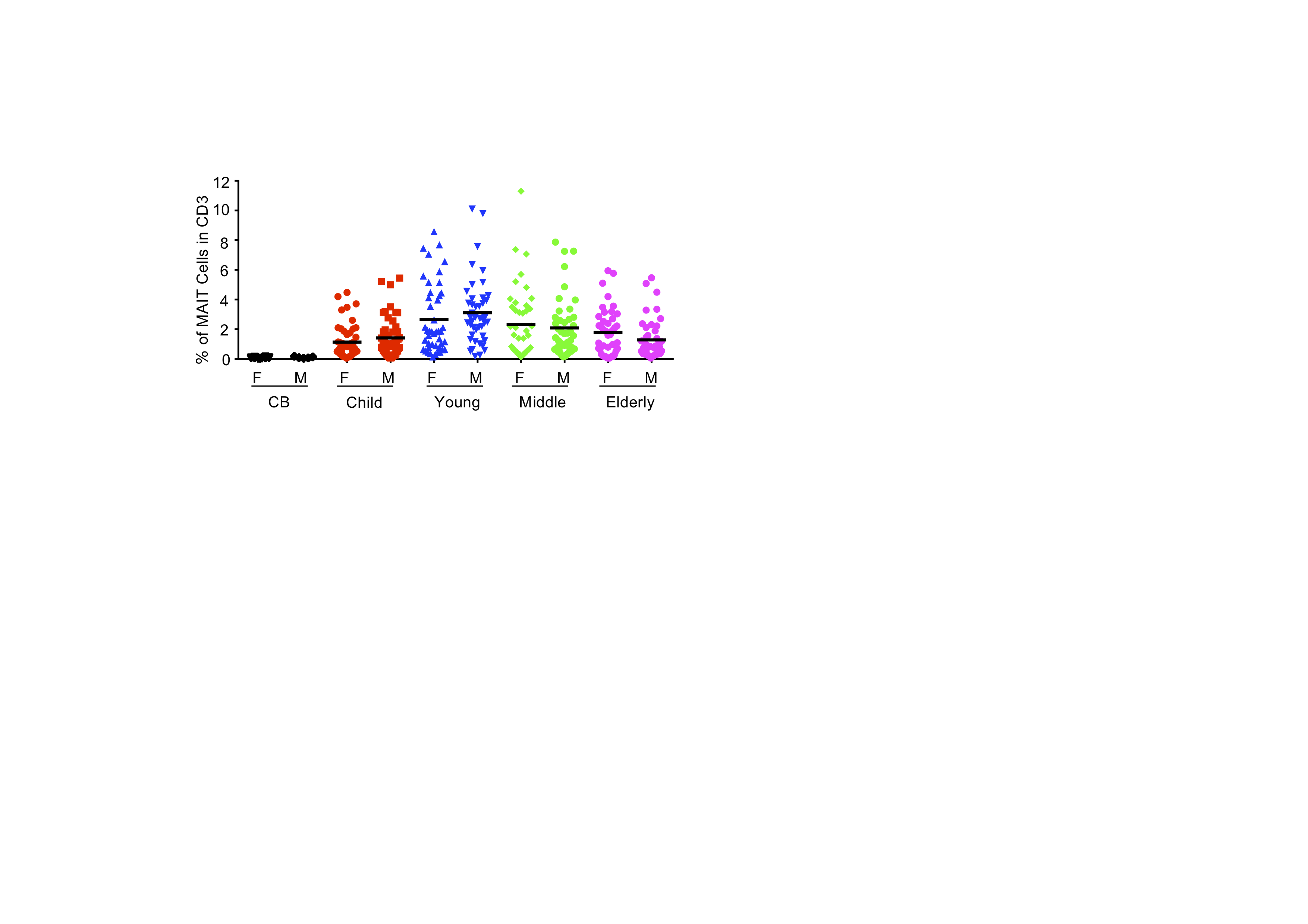

Supplement: Supplemental Figure 2 — Distribution of MAIT cell frequencies in Female (F) and Male (M) of entire groups. Freshly isolated PBMCs from 379 healthy individuals (grouped as shown in Table 1) were analyzed by flow cytometry. Each symbol represents an individual subject. Statistical significance was assessed using the Mann-Whitney U-test. Horizontal bars show the mean values, p < 0.05 were considered as statistically significant. [file Image_2.tif]
